# Immune-Mediated Colitis Induced by Immune Checkpoint Inhibitors: Pathophysiology, Clinical Management, and the Emerging Role of Fecal Microbiota Transplantation

**DOI:** 10.3390/biomedicines14030683

**Published:** 2026-03-16

**Authors:** Zeljka Belosic Halle, Vedran Tomasic, Alen Biscanin, Petra Cacic, Ivona Saric, Sanda Mustapic, Josip Stojic, Kresimir Luetic, Dinko Bekic, Matej Paic, Domagoj Micetic, Irena Krznaric Zrnic, Ivna Olic, Melanija Razov Radas, Iva Skocilic, Marin Golčic, Laura Rados, Jasna Radic, Juraj Prejac, Ivana Mikolasevic

**Affiliations:** 1 Department of Gastroenterology and Hepatology, Clinical Hospital Sveti Duh, 10000 Zagreb, Croatiakluetic@yahoo.com (K.L.); dinkobekic@gmail.com (D.B.);; 2 School of Medicine, Catholic University of Croatia, 10000 Zagreb, Croatia; tomasicvedran@gmail.com; 3 Department of Endoscopy and Day Hospital, Sestre Milosrdnice University Hospital Centre, 10000 Zagreb, Croatia; 4 Department of Gastroenterology and Hepatology, Sestre Milosrdnice University Hospital Center, 10000 Zagreb, Croatia; alen.biscanin@gmail.com (A.B.); petra.cacic@gmail.com (P.C.); 5 School of Medicine, University of Zagreb, 10000 Zagreb, Croatia; 6 Department of Gastroenterology, University Hospital Merkur, 10000 Zagreb, Croatia; 7 Department of Gastroenterology, Clinical University Hospital Dubrava, 10000 Zagreb, Croatia; smustapi2@gmail.com (S.M.); josip.stojic95@gmail.com (J.S.); 8 Department of Gastroenterology, Clinical Hospital Center Rijeka, Faculty of Medicine, University of Rijeka, 51000 Rijeka, Croatia; 9 School of Medicine, University of Rijeka, 51000 Rijeka, Croatia; 10 Gastroenterology and Hepatology Department, Internal Medicine, University Hospital of Split, 21000 Split, Croatia; ivna1211@gmail.com; 11 Division of Gastroenterology, General Hospital Zadar, 23000 Zadar, Croatia; melanijarr@gmail.com; 12 Clinic for Tumors, Clinical Hospital Center Rijeka, 51000 Rijeka, Croatia; 13 Department for Emergency Medicine of Primorsko-Goranska County, 51000 Rijeka, Croatia; 14 Department of Oncology and Nuclear Medicine, Division of Medical Oncology, University Hospital for Tumors, Sestre Milosrdnice University Hospital Center, 10000 Zagreb, Croatia; 15 Department of Oncology, University Hospital Centre Zagreb, 10000 Zagreb, Croatia; juraj.prejac@gmail.com; 16 University of Zagreb, School of Dental Medicine, 10000 Zagreb, Croatia

**Keywords:** immune checkpoint inhibitors, colitis, immunotherapy, immune-mediated colitis, microbiota, fecal microbiota transplantation, immune-related adverse events, management, cancer

## Abstract

Background/Objectives: Immune checkpoint inhibitors (ICIs) have revolutionized the treatment of various malignancies, but their use is frequently accompanied by immune-related adverse events, among which immune-mediated colitis (IMC) represents one of the most common and clinically significant gastrointestinal toxicities. IMC may lead to treatment interruption, increased morbidity, and compromised quality of life. This review aims to provide a comprehensive overview of the pathophysiology, risk factors, diagnosis, management, and emerging therapeutic strategies with particular emphasis on the role of the gut microbiota and fecal microbiota transplantation (FMT). Methods: This review integrates current international guidelines, meta-analyses, clinical trials, and recent translational studies addressing IMC. The available evidence on immunological mechanisms, predictive biomarkers, clinical presentation, diagnostic algorithms, and treatment options was critically synthesized to outline a structured and multidisciplinary management approach. Results: IMC is driven by dysregulated immune activation, cytokine release, and alterations in gut microbiota. Incidence and severity vary according to ICI class, combination regimens, tumor type, and patient-related factors. Diagnosis requires exclusion of infectious causes, laboratory assessment, and endoscopic and histologic evaluation with CTCAE-based severity grading. Corticosteroids remain the cornerstone of first-line therapy, while infliximab and vedolizumab are effective in steroid-refractory cases. Emerging therapies, including JAK inhibitors and FMT, have shown promising results in refractory disease. Conclusions: IMC is a complex and potentially severe complication of ICI therapy that necessitates early recognition, accurate grading, and individualized, multidisciplinary management. Severity-guided treatment, timely escalation to biologics, and careful balancing of immunosuppression with antitumor efficacy are essential for optimal outcomes. Future research should focus on biomarker validation, microbiome-targeted therapies, and prospective trials to refine therapeutic algorithms and define the optimal role and timing of FMT in clinical practice.

## 1. Introduction

Immune checkpoint inhibitor (ICI) therapy has revolutionized the treatment of various malignancies [1]. ICI is based on the use of monoclonal antibodies that block specific molecules functioning as immune checkpoints, thereby enhancing the host’s antitumor immune response [2]. Some of the most extensively studied immune checkpoints include programmed death-1 (PD-1) and its ligand (PD-L1), as well as cytotoxic T-lymphocyte-associated antigen 4 (CTLA-4). While immune checkpoints physiologically prevent autoimmunity, their blockade and subsequent immune stimulation can lead to autoreactive inflammatory processes in healthy tissues, known as immune-related adverse events (irAEs). These events may necessitate temporary or permanent discontinuation of anticancer therapy, which can significantly impact treatment outcomes and patient quality of life (QoL) [3].

Gastrointestinal irAEs, most notably diarrhea and immune-mediated colitis (IMC), are frequent causes of ICI therapy interruption or discontinuation [4]. Any episode of diarrhea in patients receiving ICIs warrants thorough evaluation to exclude IMC, based on comprehensive laboratory, endoscopic, and histologic assessment [5]. Gastrointestinal irAEs represent the second most prevalent category of ICI-associated toxicities after dermatologic events, with the left colon being the most commonly affected site [6]. IMC exhibits a clinical spectrum ranging from mild diarrhea to severe, life-threatening disease. Given its potential for rapid progression, early recognition, precise grading, and timely management are crucial to minimize morbidity and optimize oncologic outcomes [7,8,9,10,11].

The aim of this review is to provide a structured approach to the diagnosis, classification, treatment, and follow-up of patients with IMC, based on the latest international recommendations and available evidence.

## 2. Methods

This is a narrative, structured review of the literature focused on immune-mediated colitis associated with immune checkpoint inhibitors. A comprehensive literature search was conducted using PubMed/MEDLINE, Scopus, and Web of Science databases for articles published between January 2011 and January 2025. The search strategy included combinations of the following keywords: “immune checkpoint inhibitors,” “immune-mediated colitis,” “immune-related adverse events,” “immunotherapy,” “microbiota,” and “fecal microbiota transplantation.”

Priority was given to international clinical guidelines (e.g., ASCO, ESMO, AGA), systematic reviews, meta-analyses, randomized clinical trials, and large observational studies. Relevant translational and mechanistic studies were also included to provide insight into pathophysiology and microbiome-related mechanisms.

Articles were selected based on their relevance to the pathophysiology, risk factors, diagnosis, management, and emerging therapies for immune-mediated colitis. Case reports and small case series were included primarily in sections discussing refractory disease and fecal microbiota transplantation due to the limited availability of higher-level evidence. Non-English publications, conference abstracts without full text, and studies not directly related to immune-mediated colitis were excluded.

The evidence was qualitatively synthesized to provide a clinically oriented and multidisciplinary overview rather than a formal systematic meta-analysis.

## 3. Pathophysiology and Immunological Mechanisms

The pathophysiological mechanisms underlying IMC are not yet fully understood. Nevertheless, available evidence suggests that activation of T lymphocytes, infiltration of the colonic wall accompanied by cytokine secretion, and alterations in the gut microbiota play central roles [12,13]. CD8+ T lymphocytes of tissue-resident memory origin, through granzyme B release, appear to be crucial drivers of inflammatory activity. Evidence shows these cells seem to play an even more prominent role in IMC than in ulcerative colitis (UC). However, their absolute number has not been shown to correlate with clinical or histopathological severity of inflammation [14].

Immune mechanisms start with CTLA-4, expressed on regulatory T cells (Tregs), who interacts with CD80 and CD86 on antigen-presenting cells to suppress T-cell activation, thereby maintaining immune tolerance. Inhibition of CTLA-4 disrupts this balance, promoting CD4+ and CD8+ T-cell proliferation and cytokine release, which initiates inflammation. Similarly, PD-1, a receptor on T cells, transduces inhibitory signals upon binding to its ligand PD-L1, expressed across tumors and various immune cells. Therapeutic blockade of the PD-1/PD-L1 pathway augments antitumor immunity but concurrently promotes widespread immune activation, which may result in inflammatory injury to gastrointestinal tissues [15]. Although CD8+ T-cell activity contributes to the initiation of IMC, studies show no clear correlation between their cytotoxicity and disease severity, indicating that additional immunological mechanisms influence clinical outcomes and the need for intensified therapy [16]. Moreover, CTLA-4, highly expressed on regulatory T cells, plays a central role in maintaining tolerance; blockade of this pathway reduces Treg-mediated suppression and enhances effector T-cell activation, thereby promoting intestinal inflammation characteristic of IMC [17].

The role of the gastrointestinal microbiota in the development and severity of IMC, as well as in the therapeutic efficacy of ICIs, has been increasingly recognized [12,13]. Certain microbiome studies conducted before and after ICI administration demonstrated improved survival outcomes in patients whose gut microbiota consisted of *Faecalibacterium* and other *Firmicutes*, compared to those dominated by *Bacteroidetes* [12]. However, findings across different centers remain inconsistent and inconclusive, emphasizing the need for larger multicenter studies to provide suitable data and potentially translate microbiota profiling into clinical practice [12,14].

## 4. Risk Assessment and Predictive Biomarkers of Immune-Mediated Colitis

The incidence of IMC varies depending on the type of ICI therapy, underlying malignancy, and patient-specific factors that influence immune responsiveness to treatment. Early identification of at-risk patients and recognition of predictive biomarkers are essential to enable timely diagnosis, guide preventive strategies, and improve clinical outcomes.

### 4.1. Risk According to Type of Therapy Used

The incidence of IMC varies substantially depending on the ICI class and therapeutic regimen. Anti–CTLA-4 therapy carries a relatively high risk (8–22%), with severe IMC (Common Terminology Criteria for Adverse Events, CTCAE grade ≥ 3) developing in 5–10% of patients. In contrast, anti–PD-1/PD-L1 monotherapy is associated with a lower incidence (1–5%), and severe forms occur in up to 2% of patients. Combination ICI therapy poses the highest risk for IMC, with overall incidence rates of 15–30% and severe cases reported in 15–30% of patients [18,19,20,21]. The incidence, severity, and time to onset of diarrhea and IMC according to ICI type are summarized in Table 1.

The role of ICI dosing is not completely elucidated, although an association exists, particularly with ipilimumab. Higher-dose ipilimumab (10 mg/kg) has been associated with greater IMC risk than lower-dose regimens (3 mg/kg). Similarly, when combined with nivolumab, low-dose ipilimumab (1 mg/kg) carries lower risk than higher-dose (3 mg/kg), even with higher nivolumab dosing. By contrast, no dose-dependent differences have been observed with pembrolizumab [22,23].

### 4.2. Risk According to Cancer Type

The incidence of IMC also varies according to the underlying malignancy, ranging from 0 to 32% for all grades and 0–21% for severe cases (CTCAE grade ≥ 3) [24]. Patients with melanoma appear to have a higher risk than those with other tumor types. For instance, diarrhea develops in up to 41% of melanoma patients receiving ipilimumab, compared with 25–27% of patients treated for non–small cell lung cancer (NSCLC). Similarly, diarrhea in NSCLC patients receiving anti–PD-1/PD-L1 therapy occurs in 5–14% of cases, whereas the incidence in melanoma ranges from 10 to 22% [23]. Patients with malignancies frequently managed with dual immune checkpoint blockade (such as melanoma, NSCLC, or renal cell carcinoma) are at particularly high risk, reflecting the increased incidence associated with combination regimens [25].

### 4.3. Patient-Specific Factors

#### 4.3.1. Patients with Pre-Existing IBD

In patients with preexisting inflammatory bowel disease (IBD), irAEs following ICI therapy are markedly more common than in those without IBD. In one study, gastrointestinal irAEs occurred in 41% of patients with IBD—substantially exceeding the rates observed in non-IBD cohorts—and were frequently severe, presenting with high-grade diarrhea or colitis, a high requirement for biologic rescue therapy (infliximab [IFX] or vedolizumab [VDZ]), and occasional colonic perforation necessitating surgical intervention [26]. CTLA-4 blockade appears to confer a higher risk of gastrointestinal toxicity compared with PD-1/PD-L1 inhibition [27,28].

Elevated fecal calprotectin (FC) (>150 µg/g) and C-reactive protein (CRP), though nonspecific, may indicate subclinical inflammation preceding symptom onset [29,30].

ICI therapy may also precipitate IBD flares, making it challenging to distinguish between disease reactivation and IMC [31]. Although corticosteroids and biologics are effective in both settings, differentiating between them is clinically important.

Recent studies indicate that maintaining IBD control at baseline may reduce toxicity risk. Sleiman et al. observed that IBD patients with inactive disease or ongoing maintenance therapy had fewer gastrointestinal AE and no adverse impact on overall survival, suggesting ICIs should not be withheld when clinically indicated [32]. Similarly, Abu-Sbeih et al. reported gastrointestinal AE in 41% of IBD patients treated with ICIs, with most in remission and no deaths related to toxicity [26].

These findings emphasize the need for an individualized, multidisciplinary approach. Although risk factors for IMC in IBD patients remain unclear, optimal disease control before ICI initiation may be protective. The American Gastroenterological Association (AGA) advises that ICIs can be administered to IBD patients with caution, guided by individualized assessment and close monitoring [7]. Despite the increased risk of GI toxicity, many IBD patients can still derive meaningful oncologic benefit if irAEs are recognized and managed promptly [28].

#### 4.3.2. Patients with Preexisting Autoimmune Comorbidities

Beyond IBD, a wide spectrum of preexisting autoimmune disorders—such as rheumatoid arthritis, systemic lupus erythematosus, psoriasis, autoimmune thyroiditis, scleroderma, vasculitis, and interstitial lung disease—has been evaluated in the context of ICI therapy. Although these patients are typically excluded from clinical trials, real-world and retrospective data indicate that ICIs can be administered cautiously, with manageable risk in most cases [33,34].

Meta-analyses consistently demonstrate that patients with preexisting autoimmune diseases have approximately a twofold higher risk of developing irAEs compared with those without autoimmune comorbidity. The predominant complications include flares of the underlying autoimmune disorder and de novo irAEs, occurring in up to 40% and 30% of patients, respectively. While most flares are mild to moderate and respond well to corticosteroid therapy, 7–20% of patients ultimately require discontinuation of ICI treatment [35,36,37,38,39].

Despite these risks, oncologic outcomes—including objective response rate (ORR), progression-free survival (PFS), and overall survival (OS)—remain comparable to those of the general cancer population, suggesting that ICI efficacy is largely preserved in this setting [34,40].

Baseline immunosuppressive therapy does not appear to significantly affect these outcomes, though severe, active autoimmune disease or high-dose immunosuppression may contraindicate ICI initiation [34].

The pattern of irAEs often reflects the underlying autoimmune condition: exacerbations tend to involve the same organ system previously affected, while unrelated irAEs occur at rates similar to those in non-autoimmune patients [37]. Cai et al. further quantified this risk, reporting pooled relative risks of 1.74 for any-grade and 1.43 for severe (CTCAE ≥ 3) irAEs [41].

From a management perspective, ICIs should not be categorically withheld from patients with autoimmune comorbidities. Instead, treatment should follow an individualized approach emphasizing disease control before therapy, involvement of relevant specialists, and close monitoring for flares. Continuation or discontinuation of ICIs should be guided by the severity of adverse events, availability of effective immunosuppression, and overall oncologic prognosis [34,40].

### 4.4. Predictive Biomarkers

Identifying reliable predictive biomarkers is crucial for risk stratification, early diagnosis, and therapeutic guidance. Traditional biomarkers, such as the neutrophil-to-lymphocyte ratio (NLR), circulating tumor cells, lactate dehydrogenase, and the prognostic nutritional index, have demonstrated moderate predictive capacity for irAEs [42]. Xu et al. developed and validated a nomogram based on β2-microglobulin, globulin, NLR, and CD4/CD8 ratio for estimating IMC risk in patients with lung cancer [43].

Emerging biomarkers include cytokine signatures such as elevated IL-6 and IL-17 (IL-interleukin), which drive Th17-mediated inflammation, and soluble CTLA-4, which reflects dysregulated T-cell tolerance; all of these have been associated with an increased risk of IMC and other irAEs in patients receiving ICIs [44,45]. Neurofilament light chain and anti-neuronal autoantibodies also show potential in predicting neurological irAEs and may be relevant in enteric neuropathy-associated IMC [46]. Elevated serum amyloid A and the ATG16L1 T300A polymorphism have also been associated with more severe forms of IMC in a retrospective study by Kogan et al., while lower levels of IBD-associated antibodies to *Saccharomyces cerevisiae* (ASCA) were recorded in patients who required more intensive immunosuppressive therapy, which indicates their potential as predictive biomarkers. Other serological and inflammatory markers (ICAM-1, VCAM-1, VEGF, pANCA, anti-OmpC) did not show significant associations with outcomes [47]. Furthermore, considering the role of microRNA (miRNA) in epigenetic regulation of gene expression, several studies have investigated the diagnostic and prognostic value of miRNA in the development of solid tumors and IBD, including colitis [48,49,50]. However, as markers in predicting irAEs, they remain insufficiently known. In a pilot study, six miRNAs were identified as being significantly elevated and three as being decreased in basal samples from cancer patients who developed IMC. Moreover, some miRNA signatures were associated with a specific type of primary tumor [51].

Microbial biomarkers have received considerable attention. Enrichment of anti-inflammatory taxa like *Faecalibacterium prausnitzii* and *Akkermansia muciniphila* is associated with improved ICI response and reduced IMC incidence [52,53,54]. In contrast, depletion of *Bacteroides* spp. and reduced microbial diversity correspond with heightened toxicity risk. Metagenomic and metabolomic profiling of the gut microbiome further enables identification of functional microbial metabolites, such as short-chain fatty acids (SCFAs), as biomarkers for immune modulation [54]. Radiologic and digital biomarkers, including increased intestinal fluorodeoxyglucose (FDG) uptake on PET/CT (Positron Emission Tomography/Computed Tomography) and real-time data from wearable sensors, are being explored to anticipate IMC onset or flare [55]. Integration of these data streams using machine learning has the potential to yield powerful multimodal biomarker models [56].

## 5. Clinical Presentation, Diagnosis

Diarrhea represents the most common clinical manifestation of IMC, occurring in approximately 13–37% of patients receiving ICIs [57]. Up to 9% of patients may also present with abdominal pain, fever, rectal bleeding, or passage of blood and mucus in the stool; however, the absence of diarrhea does not exclude colitis [57,58].

Symptom onset typically occurs within the first 6–8 weeks after ICI initiation, although delayed presentations have been reported, occasionally emerging up to two years after treatment discontinuation [15,58]. The clinical spectrum ranges from mild increases in stool frequency to life-threatening colitis complicated by dehydration, sepsis, toxic megacolon, or intestinal perforation [7].

The Common Terminology Criteria for Adverse Events (CTCAE) remain the standard tool for grading IMC severity and guiding therapeutic decisions, incorporating both symptomatology and treatment response [5,7]. Grading of IMC according to CTCAE is summarized in Table 2.

A structured diagnostic approach is essential to exclude alternative etiologies of diarrhea or colitis in patients currently or previously treated with ICIs. The recommended initial work-up includes complete blood count, FC, CRP, and electrolyte and metabolic panels (urea, creatinine, potassium, sodium, calcium, magnesium, liver enzymes, and albumin). Additional testing should assess for hepatitis B and C, latent tuberculosis, and markers of malabsorption (iron, ferritin, vitamin B12, vitamin D, folic acid), as well as thyroid function and celiac serology [15,59,60].

Although most cases are ICI-induced, infectious etiologies must be excluded before establishing the diagnosis [4,9]. Stool cultures and testing for *Clostridioides difficile*, *Giardia duodenalis*, *cytomegalovirus* (CMV), and *Epstein–Barr virus* (EBV) are recommended, as these pathogens may exacerbate or resemble IMC [55].

Screening for ova and parasites should be guided by local epidemiology and exposure history [10]. In patients with fever, severe abdominal pain, or peritoneal signs, abdominal CT is warranted to rule out complications such as perforation, megacolon, or abscess formation [1,2,10]. Non-infectious conditions such as lymphocytic or collagenous colitis, anemia, electrolyte abnormalities, treatment-related thyroid toxicities, immune-mediated pancreatic insufficiency, and de novo celiac disease may clinically simulate IMC and should therefore always be considered and excluded during diagnostic evaluation [58].

Ileocolonoscopy with mucosal biopsies remains the reference standard for diagnosing IMC in patients presenting with persistent diarrhea of CTCAE grade ≥ 2 [10]. The endoscopic appearance of ICI-related inflammation is nonspecific, ranging from normal mucosa to varying degrees of edema, erythema, erosions, aphthous lesions, inflammatory exudate, and ulcerations [61]. A flexible sigmoidoscopy is often sufficient for diagnosis, as the left colon is involved in up to 98% of cases. However, certain high-risk endoscopic features—including deep ulcerations and extensive colonic involvement—are predictive of steroid-refractory disease and an increased likelihood of requiring biologic therapy [62]. Importantly, a normal-appearing mucosa does not exclude IMC, as histologic inflammation can be present even when endoscopic findings are unremarkable. Indeed, up to 37% of patients with IMC have a normal-appearing colon on endoscopy; therefore, biopsies should always be obtained regardless of mucosal appearance [20,62]. Currently, there is no specific endoscopic scoring system for IMC. In clinical practice, the Mayo Endoscopic Subscore (MES) or the MD Anderson Cancer Center classification are commonly applied to grade endoscopic severity and guide management (Table 3) [63,64].

Histologic findings are not pathognomonic. The most common histologic features of IMC are increased lamina propria cellularity, intraepithelial neutrophilic infiltrates, cryptitis, crypt abscesses, and apoptotic cells, which may precede the onset of diarrhea [20]. Differentiating IMC from IBD can be challenging due to overlapping clinical, endoscopic, and histologic features. Unlike IBD, which is typically characterized by architectural distortion, basal plasmacytosis, or granulomas, IMC more often demonstrates a mixed neutrophilic and lymphocytic infiltrate, crypt abscesses, and increased epithelial apoptosis, but generally lacks the chronic mucosal remodeling observed in longstanding IBD.

Ultimately, an accurate diagnosis relies on the integration of clinical symptoms, association with or history of ICI therapy, endoscopic and histologic findings, and the exclusion of infectious and non-infectious causes of diarrhea [65].

## 6. Management and Treatment Strategies

The objectives of treating IMC are to rapidly control symptoms and improve patients’ QoL by achieving a symptomatic response followed by clinical remission, to prevent life-threatening complications such as dehydration, ileus, toxic megacolon, intestinal perforation, septic shock, and death, and, when possible, to continue or reintroduce immunotherapy [5]. Systemic immunosuppression resulting from corticosteroids, biologics, or JAK inhibitors increases the risk of opportunistic infections and may potentially impair ICI-induced antitumor immunity. Early recognition and a stratified treatment approach according to clinical severity and response are essential to minimize morbidity without compromising oncologic efficacy.

Management of IMC is guided primarily by the severity of colitis according to CTCAE and the patient’s response to corticosteroid therapy; however, symptom duration, along with endoscopic, histologic and biochemical findings, can also be useful predictors of treatment response and outcomes [7,66]. Treatment options are comprehensively detailed in Table 4, organized by treatment line and CTCAE grade to facilitate clear clinical decision-making based on disease severity.

### 6.1. First-Line Therapy

#### 6.1.1. Mild Colitis (CTCAE Grade 1)

In patients with diarrhea (CTCAE 1), initial management strategy includes continuation of the ICI-treatment after excluding infection, including *Clostridioides difficile* and CMV, and measuring FC [7,15].

Supportive care includes oral hydration, dietary modification to reduce fiber, lactose, and caffeine intake, and antidiarrheal agents (e.g., loperamide).

If colitis symptoms persist beyond 72 h, escalate to CTCAE grade ≥ 2, or FC levels ≥ 150 µg/g, initiation of therapy with oral mesalamine or cholestyramine can be considered, followed by performance of colonoscopy [80]. The presence of any endoscopic activity (MES ≥ 1) necessitates temporary interruption of ICI therapy and commencement of oral corticosteroids.

In cases of histologically confirmed colonic inflammation, budesonide 9 mg/day with extended release is effective and may allow continuation of ICI therapy [67,80].

#### 6.1.2. Moderate to Severe Colitis (CTCAE Grade ≥ 2)

After excluding infection and completing laboratory and endoscopic workup, patients should be hospitalized if clinically indicated [5,7]. ICI therapy should be suspended or even stopped (anti-PD-1/PD-L1 permanently for grade 4; consideration of permanent discontinuation of anti-CTLA-4 for CTCAE ≥ 2).

Systemic corticosteroids are the mainstay of initial treatment for CTCAE ≥ 2 colitis, administered as oral or intravenous prednisone or methylprednisolone at 0.5–2 mg/kg/day [7,45,68]. Early initiation within five days is associated with shortened symptom duration and hospitalization [69]. Oral corticosteroid therapy is the preferred option for patients with Grade 2 colitis who are suitable candidates for outpatient care [68,69]. Intravenous corticosteroid therapy should be considered for all patients with Grade ≥ 3 colitis requiring inpatient care or in cases where oral therapy has failed.

Approximately 70% of patients achieve remission with corticosteroids alone [82,83,84]. Steroid tapering should be carefully managed over 4–6 weeks to minimize relapse risk [5,7].

Baseline corticosteroid use at the initiation of ICI therapy, as well as high-dose corticosteroid treatment following the onset of IMC, has been associated with poorer clinical outcomes [85,86]. However, due to the retrospective, observational, and exploratory design of these studies, the authors acknowledge multiple limitations and potential confounders, leaving the relationship between corticosteroid use and outcomes as one of correlation rather than proven causation. Extended corticosteroid courses pose significant risks, including serious adverse effects and increased mortality, notably due to potentially life-threatening infections [87,88,89,90,91].

Patients with Grade 2 colitis and mild endoscopic findings (MES 0–1) may be treated with low-dose corticosteroids (<10 mg prednisone equivalent dose) or de-escalate to mesalamine or budesonide once improved [92].

Rechallenge with ICIs may be considered in multidisciplinary discussion for patients in clinical remission or on ≤8 mg/day of methylprednisolone [61].

### 6.2. Second-Line Therapy

One-third of patients do not respond to IV steroids, and 34–44% relapse following the first-line therapy with steroids [93].

The presence of moderate-to-high-risk endoscopic features, including a MES of 3, ulcerations exceeding 10 mm in size, ulceration depth greater than 2 mm, presence of more than three ulcerations, and inflammatory changes extending proximal to the left colon, is significantly associated with an increased probability of corticosteroid treatment failure, escalation to biologic therapy, and prolonged inpatient hospitalization [94,95].

What is the optimal second-line therapy for steroid-refractory IMC that effectively alleviates colitis symptoms without compromising the antitumor immune response?

Management strategies are primarily based on expert opinions drawn from retrospective studies, case reports, meta-analyses and IBD therapy, with considerable variability and lack of standardization in the definitions of treatment response and follow-up periods.

To date, clinical studies have not adequately addressed the safety of immunosuppressive agents concerning tumor progression. Also, no biomarkers have been identified that reliably predict therapeutic response.

Second-line agents include IFX or VDZ following standard IBD induction regimens [5,7,68,69,96]. Both induce rapid clinical remission in approximately 88–89% of steroid-refractory cases after one or a few doses [85]. However, the corticosteroid-free clinical remission rate at 26 weeks following IFX treatment remains modest, reported at 50.9% [70].

The greatest experience has been with the use of IFX, especially in more severe forms of the disease, likely due to its rapid onset of action, long-standing clinical experience in IBD treatment, and lower treatment costs compared to VDZ because of the availability of biosimilars, particularly in resource-limited settings. VDZ, by selectively targeting the gut without causing systemic immunosuppression, represents a valid alternative, especially for patients with moderate disease and those who have comorbidities or an increased risk of infection.

When choosing between these two molecules, the clinician should in consultation with gastroenterologist carefully consider the patient’s specific characteristics, including the underlying malignant disease and comorbidities [7]. For example, pre-existing inflammatory bowel disease already treated with advanced therapeutic options, the presence of latent tuberculosis, HIV infection, hematologic neoplasms, or congestive heart failure may limit the use of IFX. It is also important to assess the expected duration of biologic therapy and the risks of systemic immunosuppression, potential infections, and malignancies, which are more pronounced with IFX. Additionally, attention should be given to other possible ICI side effects (such as ICI-induced hepatitis) that may influence the choice of biologic (hepatitis can also be a side effect of IFX), as well as the potential interference of biologics with the antitumor efficacy of ICIs—this is especially relevant for VDZ in primary gastrointestinal malignancies.

Patients who do not respond to initial biologic therapy may have their biologic class changed, for example from IFX to VDZ or vice versa [7]. Given the severity of IMC, the usual washout period is not awaited, but rather the new biologic is administered as soon as it becomes evident that the previous therapy is ineffective.

In current clinical practice, therapeutic sequencing between systemic anti-TNF agents such as infliximab and gut-selective anti-integrin therapy such as vedolizumab is guided not only by efficacy but also by patient-specific risk factors. Anti-TNF therapy may be preferred in cases requiring rapid disease control or in the presence of significant extraintestinal manifestations, whereas vedolizumab is often favored in patients with increased infection risk, prior or active malignancy, or situations where minimizing systemic immunosuppression is a priority. Therefore, individualized risk stratification plays a central role in optimizing treatment selection and sequencing.

However, a few open questions remain regarding the application of IFX and VDZ in the treatment of IMC. Namely, in all studies to date, these molecules have been administered intravenously as monotherapy, with concomitant administration of high-dose corticosteroids. The endpoints in these studies were frequently insufficiently defined. No data are currently available on the use of these agents as monotherapy or as first-line immunosuppressive treatments, approaches that could potentially reduce exposure of patients with IMC to corticosteroids and minimize associated side effects and tumor progression. Similarly, there are no available data on the potential combined prescription of intravenous IFX with an immunomodulator, which is the standard in IBD treatment, probably due to the increased risk of additional and prolonged immunosuppression. Although IFX and VDZ are now available in subcutaneous forms, data on their efficacy and safety in treating IMC via subcutaneous administration are currently unavailable. Furthermore, questions regarding therapy optimization include the possibility of dose escalation, therapeutic drug monitoring, accelerated induction, and the influence of factors such as hypoalbuminemia, all of which have not been sufficiently investigated to date.

Further insights into second-line immunosuppressive treatment for IMC are anticipated from ongoing clinical trials, including a randomized Phase 1/2 trial comparing the efficacy and safety of IFX versus VDZ in patients with corticosteroid-refractory colitis (NCT04407247); a Phase 2 trial comparing IFX to corticosteroids (NCT04305145); a Phase 3 randomized trial evaluating IFX plus corticosteroids versus corticosteroids alone (NCT05947669); and a Phase 2 randomized trial comparing VDZ to prednisolone with or without IFX (NCT04797325) [68].

### 6.3. Third-Line and Refractory Colitis

Patients with persistent or worsening symptoms despite second-line therapy should undergo a comprehensive evaluation, including repeat endoscopy and exclusion of infections such as CMV and *Clostridioides difficile*. Patients with IMC who are refractory to IFX typically develop symptoms within the first 4 weeks following initiation of ICI therapy. These patients are more likely to have a pre-existing autoimmune disease, present with higher-grade IMC (CTCAE grade ≥ 3), and have received prolonged corticosteroid therapy prior to the introduction of biologic agents [71].

Salvage treatments reported in limited series include immunosuppressants (mycophenolate mofetil; cyclosporine and tacrolimus), JAK inhibitors (tofacitinib), and biologics targeting IL-12/23 (ustekinumab), IL-6 (tocilizumab), and CTLA-4 (abatacept) [68,72,73,74,75,76,77,78,79,84,97].

Ongoing trials of etrasimod (NCT06521762), mesalamine (NCT05663775), and the probiotic formulation VSL#3 (NCT06508034) are expected to provide further insights into potential management strategies for IMC [68]. 

As a last resort, elective or emergency colectomy may be considered for patients with IMC who are refractory to medical therapy, as well as for those experiencing life-threatening complications such as toxic megacolon, bowel perforation, intra-abdominal abscess, or septic shock [5,7]. Additionally, elective colectomy was reported in a patient with active inflammatory bowel disease (IBD) when the therapeutic benefits of ICI significantly outweighed the morbidity associated with surgical intervention [98].

### 6.4. Supportive and Preventive Measures

All patients with IMC require thromboembolism risk assessment and appropriate anticoagulation [99]. Nutritional screening using NRS-2002 and SARC-F, with targeted interventions, is essential given malnutrition risk [100,101].

Screening for latent infections and updating vaccinations (pneumococcal, influenza) should occur before immunosuppression [5,7,102]. Prophylaxis against *Pneumocystis jirovecii* is recommended during high-dose corticosteroid or combined immunosuppression.

Increased dietary fiber intake beneficially modulates gut microbiome composition, particularly *Ruminococcaceae* and *Faecalibacterium* species, which has been associated with enhanced anti–PD-1 therapy efficacy [81,103]. However, the use of commercially available probiotics has not demonstrated significant microbiome alterations or improved clinical outcomes in patients receiving ICIs.

To prevent osteopenia and osteoporosis, it is essential to ensure adequate daily intake of calcium and vitamin D, promote smoking cessation, and encourage regular physical activity tailored to individual capacity. Vitamin D and calcium supplementation is indicated for all patients receiving corticosteroid therapy, while bisphosphonate treatment is recommended for those with postmenopausal or corticosteroid-induced osteoporosis [104]. A retrospective study demonstrated that the use of over-the-counter vitamin D supplements in melanoma patients treated with ICI (anti–PD-1, anti–CTLA-4, or their combination) was associated with a reduced risk of developing IMC [105]. The use of multivitamin supplements is justified only in IMC patients with documented clinically significant deficiencies of vitamins, minerals, or trace elements [103].

**Figure 1:**
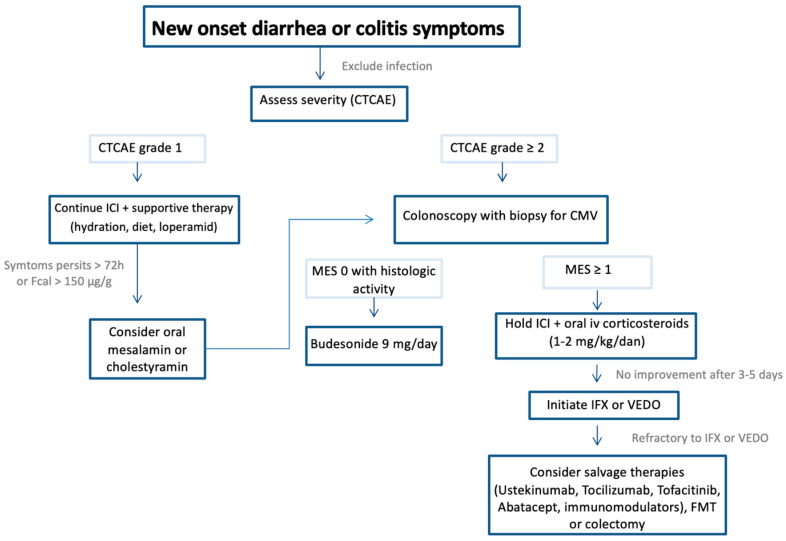
Therapeutic approach to IMC.

The treatment algorithm is shown in Figure 1.

Key challenges for future therapeutic strategies include optimizing biologic sequencing, evaluating subcutaneous formulations, defining targets for therapeutic drug monitoring, and balancing immunosuppression with antitumor efficacy. Prospective trials are required to refine induction protocols, establish robust clinical endpoints, evaluate novel agents, and explore microbiome-based interventions.

## 7. Future Directions and Emerging Therapies

### 7.1. Personalized Approaches

The implementation of personalized strategies in cancer immunotherapy extends beyond tumor genomics to encompass host immune profiling, pharmacogenomics, and microbiome features. Artificial intelligence (AI)-driven decision-support tools now incorporate patient-specific data from transcriptomics, cytokine levels, T-cell phenotyping, and gut microbiota to forecast IMC development and guide treatment decisions [56]. 

Pharmacogenomic markers, such as HLA-DQA1*02:01 and UGT1A1 polymorphisms, offer insights into susceptibility to immunotherapy-related toxicity [106,107]. Immune cell profiling—including CD8+ tissue-resident memory cells and regulatory T cells—also contributes to stratifying patients by risk and tailoring anti-inflammatory therapies [108].

Liquid biopsies, which measure circulating tumor DNA and exosomal RNA can noninvasively monitor treatment response and detect early molecular changes predictive of irAE onset [109]. Similarly, AI-guided histological analyses and digital pathology tools are gaining ground for automated scoring of inflammation in colonic biopsies [110]. In UC, a condition closely resembling IMC, AI models have predicted therapeutic outcomes with high accuracy by integrating transcriptomic data and immune cell landscapes [111]. These methodologies are now being translated to cancer care, improving precision and real-time adaptability of immunotherapeutic regimens.

### 7.2. Fecal Microbiota Transplantation

The gut microbiota, and its modulation, is proven to play an important role in immune response, antitumor response and response to immunotherapy [112,113,114]. Fecal microbiota transplantation (FMT) is a medical procedure which involves administration of donor fecal matter into the recipient’s gastrointestinal tract, either via colonoscopy or nasojejunal tube. The main goal of FMT is modulating the recipient’s intestinal microbiota. While it is generally safe, FMT may induce adverse effects, mostly mild, such as nausea, vomiting, bloating, abdominal discomfort and diarrhea [115]. The most common indication for FMT is curing recurrent *Clostridiodales difficile* infection after failure of antibiotic therapy in immunocompetent adults, but there is emerging evidence for FMT being a useful therapeutic option in other conditions including cancer [52,116,117,118]. In recent years, FMT has shown promising results as a potential therapeutic option for patients with IMC refractory to standard immunosuppressive therapy. Although corticosteroids are usually given as a 1st line IMC treatment, followed by 2nd line IFX or VEDO, there is a subset of patients which are refractory to standard therapy [119]. Up to 41% of patients with grade ≥ 2 IMC are refractory to corticosteroids but respond to IFX, while up to 11% of patients are refractory to both corticosteroids and IFX [69].

To date, scarce case reports and studies have been published on the use of FMT in 1st and 2nd line refractory IMC. The first ever report, published by Wang et al. in 2018 [120], described two patients with IMC unresponsive to 1st and 2nd line treatment who both achieved complete clinical remission following FMT. One patient achieved remission after a single FMT, while the other achieved remission after 2 applications of FMT. All stools were by the same donor. Analysis of microbiota showed similarity to donor stool post FMT, although the similarity was reduced over time. Nonetheless, an abundance of *Bifidobacterium* genera in both recipients and *Blautia* genera in one recipient were found post FMT [120]. A subsequent case report by Fasanello et al. in 2020 confirmed a similar result with patient in IMC remission following a single course of FMT after failure of treatment with corticosteroids, IFX, mycophenolate mofetil, mesalamine and VDZ [121]. In 2023, a larger case series by Halsey et al. observed clinical improvement in 10 out of 12 patients with IMC, out of which 7 patients reached full remission and 3 patients reached partial remission. They used 4 healthy donors and most patients responded to a single course of FMT, although one patient responded only after 2 courses of FMT, while the two patients did not respond even with two courses of FMT. Microbiome was analyzed and shown that responders demonstrated an increased alpha-diversity and had an increase in beneficial genera, such as *Bifidobacterium* and *Collinsella* [122]. Most recently, preliminary findings from a large study by Wang et al. involving 62 patients further support these outcomes. Their results show 80% patients achieved clinical improvement, though mild and transient adverse events were reported in 37% cases [123].

Although FMT is for now considered as a 3rd line salvage option for refractory IMC, there is a currently ongoing clinical trial by Wang et al. assessing the safety of FMT as a 1st line treatment for IMC. Out of their 12 patients so far, 75% had remission and only two patients required immunosuppression after FMT failure [124].

Collectively, these results suggest that FMT is a promising salvage strategy for patients with refractory IMC, capable of reestablishing microbial balance and mitigating intestinal inflammation. However, its optimal timing, donor selection, route of administration, and long-term effects remain to be determined, there is a need for prospective trials to define the answers to these questions. FMT studies and results are summarized in Table 5.

### 7.3. Preventive and Combined Strategies

Preventive strategies to mitigate IMC and other irAEs are of paramount importance to avoid treatment discontinuation and preserve QoL.

FMT is also being investigated as a preventive measure. Administering FMT or prebiotics before ICI initiation in patients with dysbiosis may prime a more favorable immunologic baseline. Jiang et al. showed that specific microbial signatures before treatment correlated with ICI response, suggesting the feasibility of microbiota-guided patient stratification [111].

Antibiotic stewardship remains essential, as antibiotic-induced dysbiosis negatively affects ICI efficacy and increases irAE incidence [125].

Dietary interventions and next-generation probiotics designed to enhance SCFA-producing bacteria may further prevent IMC [54].

Beyond microbiota modulation, combined therapeutic strategies offer promising avenues. Co-administration of ICIs with cytokine inhibitors (e.g., anti-IL-6, anti-IL-17), vascular endothelial growth factor (VEGF), or mitogen-activated extracellular signal-regulated kinase (MEK) inhibitors has been shown to enhance efficacy but also necessitates careful toxicity management [126,127].

Other novel approaches include extracorporeal photopheresis, dendritic cell-based vaccines, neoantigen-based vaccines, and oncolytic viruses, all of which can synergize with ICIs while offering potential immune modulation [128,129,130]. Implementation of these approaches should be individualized, biomarker-guided, and monitored longitudinally using digital health tools and remote patient-reported outcome platforms [56].

Lastly, the interactions between chronic liver disease and gut microbiota, highlighted by Stojic et al., offer translational insights. The dysbiotic changes observed in patients with liver disease, such as reduced *Faecalibacterium* and increased *Enterobacteriaceae*, parallel microbiota shifts in IMC, reinforcing the relevance of the gut-liver axis in systemic immune regulation [131].

Despite many studies, noting that a significant amount of data comes from retrospective and time-limited studies, to date, no single biomarker has proven reliable for predicting or differentiating IMC in clinical practice.

Ultimately, an integrated approach combining predictive biomarkers, personalized risk assessment, and preventive interventions such as FMT or cytokine modulation may offer the most effective strategy for managing and mitigating IMC and other irAEs. The challenge moving forward will be implementing these advances into routine clinical workflows, supported by prospective trials and real-world validation.

## 8. Oncological Outcomes After ICI Discontinuation

Discontinuation of ICIs occurs for several reasons, including irAEs, achievement of durable tumor response, patient preference, or disease progression. The long-term consequences of treatment cessation remain an active area of investigation.

Several meta-analyses have evaluated outcomes after ICI discontinuation. Pala et al. reported that long-term progression-free survival (PFS) varies depending on tumor type and reason for cessation—patients with melanoma or those treated with combined anti–PD-1 and anti–CTLA-4 therapy demonstrated more favorable outcomes, whereas poorer results were observed in renal cell carcinoma (RCC) and non–small cell lung cancer (NSCLC) when discontinuation occurred due to toxicity [132].

Tzeng et al. examined treatment-free survival (TFS) in metastatic RCC and found that 26% of patients maintained durable responses after treatment cessation, with higher TFS in those receiving dual ICI therapy compared with ICI plus VEGF-directed regimens [133].

In a meta-analysis of advanced melanoma, Mayer et al. found that both PFS and overall survival (OS) remained high for up to three years post-discontinuation. Elective cessation yielded better one-year PFS (≈91%) than discontinuation due to toxicity (≈79%), and longer treatment duration was associated with improved outcomes [134].

Lellas et al. similarly reported that approximately 21% of patients relapsed after ICI withdrawal, but time to progression was longer in those who achieved a complete response (CR), suggesting durable antitumor immunity [135].

Across tumor types, patients achieving CR prior to discontinuation consistently exhibit the most favorable long-term outcomes. Even when therapy was stopped because of toxicity, durable responses have been observed, supporting the concept of persistent immunologic memory after ICI withdrawal [136,137,138].

Rechallenge following prior discontinuation has emerged as a feasible strategy in selected patients. Cai et al. and pooled analyses across solid tumors reported objective response rates of 20–22% and disease control rates around 50% upon ICI retreatment, with incidences of all-grade and high-grade irAEs (41% and 13%, respectively) comparable to initial exposure [139,140]. These data suggest that ICI rechallenge can restore antitumor activity without a disproportionate increase in toxicity risk.

Overall, current evidence indicates that discontinuation does not necessarily compromise oncologic outcomes—particularly when ICIs are stopped after a period of disease control [134,137,141,142,143]. However, significant heterogeneity among tumor types, treatment durations, and reasons for discontinuation underscores the need for prospective trials and standardized clinical guidelines.

Patients with preexisting IBD and autoimmune comorbidities remain at higher risk for immune-related toxicity during ICI therapy. Although adverse events in these populations are more frequent and sometimes severe, they are often manageable, and therapeutic efficacy is generally preserved. Importantly, discontinuation of ICIs, whether due to toxicity or sustained tumor response, does not necessarily predict poor prognosis; durable remissions can occur even after treatment withdrawal.

Future research should focus on identifying predictive biomarkers that define which patients are at greatest risk for autoimmune complications and which can safely discontinue therapy without compromising long-term disease control. Until such data are available, multidisciplinary collaboration, individualized decision-making, and early intervention remain the cornerstones of optimizing safety while maintaining oncologic benefit.

## 9. Conclusions

IMC represents one of the most significant gastrointestinal toxicities associated with ICI therapy, with a broad clinical spectrum ranging from mild diarrhea to life-threatening colitis. As the use of ICIs continues to expand across oncological indications, the incidence and clinical relevance of IMC are expected to increase, underscoring the need for standardized diagnostic and therapeutic strategies.

Current evidence indicates that IMC is driven by complex interactions between immune activation, loss of peripheral tolerance, cytokine dysregulation, and alterations in the gut microbiota. Although corticosteroids remain the mainstay of first-line treatment, a substantial proportion of patients require escalation to biologic therapy, most commonly infliximab or vedolizumab, highlighting the need for early risk stratification and timely intervention. Importantly, treatment decisions must balance effective control of inflammation with preservation of antitumor immune responses and overall oncologic outcomes.

Emerging data suggest that predictive biomarkers, including inflammatory markers, cytokine signatures, microbiome composition, and multimodal digital biomarkers, may enable earlier identification of high-risk patients and more personalized management. Microbiome-targeted interventions, particularly fecal microbiota transplantation, are gaining attention as promising salvage and potentially preventive strategies in refractory IMC, although robust prospective data are still lacking.

## Figures and Tables

**Table 1:** Incidence of diarrhea and IMC based on the type of ICI used.

ICI	Incidence of Diarrhea (CTCAE 1–4)	Incidence of IMC (CTCAE 1–4)	Severe IMC (CTCAE ≥ 3)	Time to Onset (Average)
anti-CTLA-4	30–35%	~8–22%	5–10%	~6–7 weeks
anti-PD-1/PD-L1	12–14%	~1–5%;	1–2%	~1 week–2 years
anti-PD-1/(L)1 + anti-CTLA-4	up to 40%	15–30%	10–15%	~7 weeks

ICI, Immune Checkpoint Inhibitors; CTCAE, Common Terminology Criteria for Adverse Events; CTLA-4, Cytotoxic T-Lymphocyte–Associated Antigen 4; PD-1, Programmed Death-1; PD-L1, Programmed Death Ligand-1.

**Table 2:** Grades of IMC according to CTCAE.

	Grade 1	Grade 2	Grade 3	Grade 4	Grade 5
**Diarrhea**	Increase of <4 stools/d over baseline; mild increase on ostomy output compared with baseline	Increase of 4–6 stools/d over baseline; moderate increase in ostomy output compared with baseline; limiting instrumental ADL	Increase of ≥7 stools/d over baseline; hospitalization indicated; severe increase in ostomy output compared with baseline; limiting self-care ADL *	Life-threatening consequences, urgent intervention indicated	Death
**Colitis**	Asymptomatic; clinical or diagnostic observations; intervention not indicated	Abdominal pain; mucus or blood in stool	Severe or persistent abdominal pain; fever; ileus; peritoneal signs	Life-threatening consequences: urgent intervention indicated	Death

* ADL, activities of daily living.

**Table 3:** MD Anderson Cancer Center Endoscopic Inflammation Grading.

Severity	Endoscopic Features
Mild	Normal endoscopy and normal histology
Moderate	Normal colon appearance with pathology showing inflammation; small ulcers <1 cm, shallow ulcer <2 mm, and/or number of ulcers <3; inflammation limited to the left colon only, no ulcer inflammation
High	Large ulcer ≥1 cm, deep ulcer ≥2 mm, and/or number of ulcers ≥3; extensive inflammation beyond left colon

**Table 4:** Medications for IMC: Dosage, Duration, and Response.

Agent/Intervention	Dosing/Administration	Timing/Duration	Response Rates	Key Considerations/Contraindications
Corticosteroids [5,7,45,67,68,69]	Oral prednisone/methylprednisolone 0.5–1 mg/kg/day (CTCAE 2; outpatient care) IV methylprednisolone 1–2 mg/kg/day (CTCAE ≥ 3 or insufficient response on/relapse off oral corticosteroids; inpatient care) Oral budesonide MMX 9 mg/day (microscopic colitis)	4–6 weeks	70%	Response assessment at day 3–5; taper over 4–6 weeks. Increased risk of AE, serious infections and mortality. May impair antitumor ICI induced immune response. Relapse rates 34–44%.
Infliximab [5,7,70,71]	Intravenous 5 mg/kg (week 0, repeat as needed on week 2 and 6; maintenance every 8 weeks)	6 weeks induction; maintenance as indicated	88% clinical remission; 50.9% corticosteroid-free remission at week 24	Fast acting (response within 7–14 days expected). Moderate to severe steroid-refractory disease (especially if risk of complications); increased risk of serious infections and may impair antitumor ICI induced immune response. Biosimilars available. No data on sc formulation, dose escalation, TDM, combination therapy with immunomodulators.
Vedolizumab [5,7,70]	Intravenous 300 mg (week 0, repeat as needed on week 2 and 6; maintenance every 8 weeks)	6 weeks induction; maintenance as indicated	89% clinical remission	Moderate steroid or IFX refractory disease. Gut selective without systemic immunosuppression. Slower onset of action (>14 days); use in severe cases with caution. No biosimilars available. No data on subcutaneous formulation, dose escalation, therapeutic drug monitoring.
Tofacitinib [72,73,74]	Oral 2 × 10 mg/day	4–25 weeks	Clinical response within 5 days; clinical, biochemical, endoscopic and histologic remission at week 5–6.	Case reports in IFX refractory disease. Thromboembolism risk assessment needed. Generally, not recommended for elderly.
Ustekinumab [74,75,76]	Single IV induction dose according to body weight (260 mg for <55 kg; 390 mg for 55–85 kg; 520 mg for >85 kg) followed by 90 mg sc dose every 8 weeks after induction dose	>6 months	Clinical remission at with significant improvement in endoscopy after 6 months	Case reports in refractory colitis. Favorable and established long-term safety profile in IBD literature.
Tocilizumab [77]	IV 8 mg/kg every 4 weeks	20 weeks	At week 24, 84% had ≥1 grade steroid-free symptom reduction	Median time to response 14 days. Results from open-label clinical study. 25% of patients had serious treatment related AEs.
Mycophenolate mofetil [78]	Oral enteric-coated 2 × 500–1000 mg/day	N/A	44% clinical remission in IFX refractory IMC.	Concomitant with corticosteroids hastens improvement of colitis or reduce the incidence of relapse.
Cyclosporine/Tacrolimus [74,79]	Oral cyclosporine 5 mg/kg in a split twice-daily dosing or IV cyclosporine 2 mg/kg/day	54 days	72.7–74% of patients responded to calcineurin inhibitor in IFX refractory IMC	Case series. Trough levels 100–200 ng/mL. Risk of renal impairment development.
Mesalamine [67]	Oral; dosing N/A	241 days		May be effective for patients with delayed onset of CTCAE 1–2 colitis and no ulcerations on endoscopy.
Loperamide [5,7]	Oral; up to 16 mg/day	Up to 72 h		For CTCAE 1 colitis. Caution in malnourished and moderately to severely dehydrated patients.
Cholestyramine [80]	Oral; dosing N/A	241 days		May be effective for patients with delayed onset of Grade 1–2 colitis and no ulcerations on endoscopy.
Probiotics [81]	Commercially available probiotics (strains and dosing were not specified)	N/A	N/A	No significant microbiome alterations or improved clinical outcomes in patients receiving ICI for melanoma.

IFX, infliximab; TDM, therapeutic drug monitoring; IV, intravenous.

**Table 5:** FMT studies summary: methodology and results.

Study	Design	Patients	Prior Treatment	Donor	FMT Route	Number of FMTs	Clinical Outcomes	Adverse Effects	Microbiome Findings
[120]	Case series	2	Steroids Infliximab	Single healthy donor	Colonoscopy	1 (patient 1) 2 (patient 2)	Complete clinical and endoscopic remission	Mild, self-limited GI symptoms	Shift toward donor-like microbiota; enrichment of Bifidobacterium spp. (both) and Blautia spp. (one); increased mucosal Tregs
[121]	Single case report	1	Steroids Infliximab Vedolizumab Mycophenolate mofetil Mesalamine	Healthy donor	Colonoscopy	1	Complete clinical remission	No serious adverse events	Not reported
[122]	Multicenter case series	12	Steroids Infliximab Vedolizumab	4 healthy donors	Colonoscopy	Mostly 1, 3 patients 2	Clinical improvement 10/12 (83%); complete remission 7 (58%); partial 3 (25%); 2 non-responders	Mostly mild transient symptoms	Shift toward donor-like microbiota, increased alpha-diversity; enrichment of Bifidobacterium and Collinsella
[123]	Prospective observational study	62	Immunosuppressive therapy	Not reported	Not reported	Not reported	Clinical improvement ~80% (patient-reported outcomes)	Mild transient adverse events ~37%	Not reported
[124]	Ongoing prospective trial (first-line FMT)	12	None	Not reported	Not reported	Not reported	Remission 75%; 2 required subsequent immunosuppression	No major safety concerns reported	Not reported

## Data Availability

No new data were created or analyzed in this study.

## Abbreviations

The following abbreviations are used in this manuscript:

AI	Artificial Intelligence
ASCA	Anti-Saccharomyces Cerevisiae Antibodies
CD	Crohn’s Disease
CMV	Cytomegalovirus
CR	Complete Response
CRP	C-Reactive Protein
CT	Computed Tomography
CTCAE	Common Terminology Criteria for Adverse Events
CTLA-4	Cytotoxic T-Lymphocyte-Associated Antigen 4
DCR	Disease Control Rate
FC	Fecal Calprotectin
FMT	Fecal Microbiota Transplantation
HLA	Human Leukocyte Antigen
IBD	Inflammatory Bowel Disease
ICAM-1	Intercellular Adhesion Molecule-1
ICI	Immune Checkpoint Inhibitor
IFX	Infliximab
IL	Interleukin
IMC	Immune-Mediated colitis
imDC	Immune Mediated Diarrhea and Colitis
irAEs	Immune-Related Adverse Events
JAK	Janus Kinase
LDH	Lactate Dehydrogenase
MEK	Mitogen-activated Extracellular signal-regulated Kinase
MES	Mayo Endoscopic Subscore
miRNA	MicroRNA
NLR	Neutrophil-to-Lymphocyte Ratio
NRS-2002	Nutrition Risk Screening 2002
NSAID	Nonsteroidal Anti-Inflammatory Drugs
NSCLC	Non-Small Cell Lung Cancer
OmpC	Outer Membrane Protein C
OS	Overall Survival
pANCA	Perinuclear Anti-Neutrophil Cytoplasmic Antibody
PD-1	Programmed Death-1
PD-L1	Programmed Death-Ligand1
PET/CT	Positron Emission Tomography/Computed Tomography
PFS	Progression-Free Survival
PPI	Proton Pump Inhibitor
RCC	Renal Cell Carcinoma
SARC-F	Sarcopenia Assessment Questionnaire
SCFA	Short-Chain Fatty Acid
Tregs	Regulatory T Cells
TFS	Treatment-Free Survival
UC	Ulcerative Colitis
UGT1A1	UDP-Glucuronosyltransferase 1A1
VCAM-1	Vascular Cell Adhesion Molecule-1
VDZ	Vedolizumab
VEGF	Vascular Endothelial Growth Factor
QoL	Quality of Life
